# Diversity and Abundance of Soil Collembola during GM Rice Overexpressing Cry1B-Cry1Aa Cultivations at Four Confined Field Trials in West Java

**DOI:** 10.21315/tlsr2022.33.3.6

**Published:** 2022-09-30

**Authors:** Yayuk Rahayuningsih Suhardjono, Amy Estiati, Syamsidah Rahmawati, Satya Nugroho

**Affiliations:** 1 Research Centre for Biology-Indonesian Institute of Sciences, Jl. Raya Bogor Km 46, Cibinong, Kabupaten Bogor 16911, Indonesia; 2 Research Centre for Genetic Engineering, National Research and Innovation Agency (BRIN), Jl. Raya Bogor Km 46, Cibinong, Kabupaten Bogor 16911, Indonesia

**Keywords:** *Bt* rice, Cry1B-Cry1Aa, Indonesia, Collembola, Diversity and Abundance

## Abstract

*Collembola* (springtails) is an important soil biology indicator to monitor toxicity or ecological disturbances in the ecosystem. The impact of *Bacillus thuringiensis (Bt)* rice cv Rojolele events expressing Cry1B-Cry1Aa driven by the maize ubiquitin promoter resistant to yellow rice stem borer (YSB, *Scirpophaga incertulas* Walker) on non-target Collembola community was assessed. The experiment was performed at four locations under confined field trials according to the Indonesia’s environmental safety regulation on genetically engineered crops. Six transgenic rice events were tested with non-transgenic Rojolele and the moderately resistant IR42 rice varieties as controls. The experimental design was randomised block design with three replicates. Collembola were collected from the bunds between plots using pitfall and Berlese funnel traps at seedling, vegetative and generative stages, as well as at harvesting time. The results showed that Collembola abundance and diversity were significantly affected by both experimental sites and observation times. However, no significant differences in Collembola diversity and abundance between *Bt* rice and non-*Bt* controls were observed. Thus, we can conclude that the cultivation of the *Bt* rice cv Rojolele events expressing Cry1B-Cry1Aa protein fusion do not adversely affect biodiversity and abundance of Collembola at the four confined rice fields.

**Highlights**
- Six transgenic *Bt* rice events cv Rojolele harbouring the *cry1B-cry1Aa* fusion genes (Rjl 04.F2.2 2.4-25-22-12-3-22, RFZ 3.2.2-1-6-28-1-10, RFZ 3.3.2A-11-25-12-5-3, RFZ 4.2.2-1-27-13-6-7, RFZ 4.2.3-28-15-2-8-20, and RFZ 4.2.4-21-8-16-7-8), non-transgenic Rojolele and the moderately resistant IR42 rice varieties, were tested for their impact on biodiversity and abundance of Collembola in four locations.
- Collembola abundance and diversity were significantly affected by both experimental sites and observation times, but no significant differences were observed in Collembola diversity and abundance between *Bt* rice and non-*Bt* controls.
- Cultivation of the *Bt* rice cv Rojolele events expressing Cry1B-Cry1Aa protein fusion do not adversely affect biodiversity and abundance of Collembola at the four confined rice fields.

## INTRODUCTION

The rice yellow stem borer (YSB, *Scirpophaga incertulas* Walker) is one of the most economically damaging insect pests in the rice field in Indonesia. Due to the unsustainability and detrimental effects on health and environment of widely used chemical to control rice YSB, breeding approaches to improve rice resistance to this economically important Lepidopteran is urgently required. Unfortunately, no resistance gene against rice YSB has been identified and mapped in rice (*Oryza sativa*) and its wild relative’s gene pools which impedes the development of resistant rice varieties through conventional breeding (Makkar & Bentur 2017). Genetic engineering approach, therefore, is an important alternative to accelerate the development of rice resistant varieties.

*Bt* toxins encoded by *cry* genes of *Bacillus thuringiensis* (*Bt*) have been reported to be highly toxic to and effectively control insects belong to the Lepidopterans, Coleopterans and Dipterans, but non-toxic to human and other animals (Bravo 1997; Makkar & Bentur 2017). *Bt* rice lines developed using genetic engineering approach containing either a single, fusion, or stacked *Bt* genes have been shown to be highly toxic and excellent for managing rice YSB in the field and thus could potentially reduce the application of chemical insecticide (Breitler *et al*. 2004; Chen *et al*. 2010; Ho *et al*. 2006; Liu *et al*. 2016). Today, *Bt* crops; i.e., corn, cotton, potato, tobacco, sugarcane and eggplant, have been grown and produced commercially in many countries around the globe (International Service for the Acquisition of Agri-biotech Applications [ISAAA] 2018).

Biosafety, which include environment and food/feed safeties, are required for the release of GM crops depending on each country’s regulation (Estiati & Herman 2016; Kumar 2014; Prakash *et al*. 2011). Previous reports on the safety assessment of Cry toxin impacts to biodiversity and non-target organisms (NTO’s) were mainly on the Cry1Ab and Cry1Ac toxins, whereas other Cry toxins have not been widely studied (Mendelsohn *et al*. 2003). Reports on the effect of *Bt* rice, including Indica rice (Cry1Ab/Ac) against rice leaf folder (*Cnaphalocrocis medinalis*) and Elite Vietnamese rice (Cry1Ab-1B and hybrid *Bt* gene Cry1A/Cry1Ac) against rice YSB in 76 different risk assessment experimental trials, on the NTO’s including beneficial insects, natural pest controllers, rhizobacteria, growth promoting microbes, pollinators, soil dwellers, aquatic and terrestrial vertebrates, mammals and human, have been performed (Yaqoob *et al*. 2016). The results showed that *Bt* crops have no significant harmful effect on NTO’s.

*Bt* rice lines cv Rojolele overexpressing fusion Cry1B-Cry1Aa proteins under the maize constitutive Ubiquitin promoter, with improved resistance to rice YSB, have been developed using *Agrobacterium* mediated transformation (Nugroho, Sari, *et al*. 2021; Nugroho, Estiati, *et al*. 2021; Rahmawati & Slamet-Loedin 2006; Usyati *et al*. 2009). According to Indonesia’s safety regulation on genetically modified (GM) products, a newly generated GM crops, in addition to food and feed safeties, must pass the environmental safety assessment to ensure that there are no adverse impacts on biodiversity and abundance of NTO’s within and around cultivation fields upon the release of GM crops to the environment (Estiati & Herman 2016).

One of the soil organisms that are found abundantly in the rice field is Collembola. Along with Acari (mites), it is the dominant soil microarthropods in terms of abundances and biodiversity (Lavelle & Spain 2001). While Acari dominates forest soils and undisturbed habitats, Collembola is important in managing grasslands and especially in arable lands, such as rice fields ecosystem (Filser 2002). There were reports that along with Chironomids and Ephydrid flies, Collembolas represent 28% of the total abundance of Arthropods collected from 12 locations of rice fields in Java (Settle & Whitten 2000).

Collembolas (springtails) are essentials for soil health by playing roles in decomposing and distributing organic materials in soil while increasing its physical properties and fertility (Indriyati & Wibowo 2008), and are important for soil nitrogen and carbon cycling (Filser 2002). Most Collembolas feed on decaying material, fungi and bacteria, and others feed on arthropod feces, pollen, algae, and other materials. Collembola play important roles in food chains and served as an alternative food for natural enemies of important crop pests (Suhardjono *et al*. 2012) and in fact they closely interact with all elements of the decomposer food web (Lee & Widden 1996; Visser 1985). They are also active under most environmental conditions (Filser 2002).

Collembola covers seven families (Poduridae, Hypogastruridae, Onychiuridae, Isotomidae, Entomobryidae, Neelidae and Sminthuridae). Due to their abundance, diversity and important roles in the environment, Collembola can be used as bioindicators in monitoring an ecosystem (Suhardjono *et al*. 2012). In this experiment, we monitored the impact of the cultivation of six transgenic rice events cv Rojolele expressing the fusion proteins Cry1B-Cry1Aa on the diversity and abundance of Collembola at four confined field trials in West Java, Indonesia. The monitoring was performed throughout the life span of the rice growth, from the seedling stages until harvesting time.

## MATERIALS AND METHODS

### Ethic Statement

Confined field trials were conducted at four different locations (Sukamandi, Muara, Banten and Kuningan) in West Java, Indonesia from 2012 to 2013. These trials were performed following the “Indonesian Guidelines for the Implementation of Biosafety Testing of Genetically Engineered Agricultural Biotechnology Products: Plant Series” and was approved by the Technical Team for Environmental Biosafety of Genetically Modified Product of the Republic of Indonesia. No vertebrates, protected or endanger species were included.

### Plant Materials

Six transgenic single insertion rice events harbouring the *cry1B*-*cry1Aa* fusion gene (Rjl 04 F2.2 2.4-25-22-12-3-22(A), RFZ 3.2.2-1-6-28-1-10(B), RFZ 3.3.2A-11-25-12-5-3(C), RFZ 4.2.2-1-27-13-6-7(D), RFZ 4.2.3-28-15-2-8-20(E), and RFZ 4.2.4-21-8-16-7-8(F)) were used. Wild-type parental rice cv Rojolele, with (G) or without pesticide application (H), and IR 42 (I) were used as susceptible controls.

### Rice Planting and Management

Rice handling, planting and plot design was carried out according to the “Indonesian Guidelines for the Implementation of Biosafety Testing of Genetically Engineered Agricultural Biotechnology Products: Plant Series”. Field experiment in Sukamandi (13 m above sea level) and Muara (259 m above sea level) were started from June to December 2012, whereas in Banten (6 m above sea level) and Kuningan (447 m above sea level) were started from May to December 2013. Each lines were planted on 10 m × 8 m experimental plots with distance between plots of 0.5 m, and spacing of 25 cm × 25 cm in a randomised block designed with three replications. Fertiliser application and weeding were applied according to the recommendations. Chlorantraniliprole based insecticide (Prevathon, Dupont) was applied at intervals of 2 weeks, starting at 2 weeks after planting to 2 weeks before harvest at a dose of 0.5 L Ha^−1^ (concentration 2 mL^−1^) in plots G of rice cv Rojolele with pesticide application.

### Collembola Sampling

Collembola was collected using both pitfall trap and modified Berlese funnel to capture surface-active (epedaphic) and soil dwelling (euedaphic) Collembola, respectively. The traps were placed alternately with a distance of 1.5 m on the bunds between plots planted with the same lines of *Bt* or non-*Bt* (Suhardjono *et al*. 2012). Specimens were collected at seedling, vegetative, and generative (flowering) stages, as well as at harvesting time. Collembola specimens were identified and classified to the level of genus except for family of Tomoceridae following Collembolans classification (Suhardjono *et al*. 2012) and counted at Laboratory of Zoology, Research Centre for Biology-LIPI.

### Data Analysis

The diversity of Collembola indicated by total genus in the habitat was counted by Shannon diversity formula (Ludwig & Reynolds 1988):


$${\text{H}}^{\prime }=\Sigma_{n=1-s}^{3}\left[\left(\frac{ni}{n}\right)\times \text{ln }\left(\frac{ni}{n}\right)\right]$$

where, H′ = Shannon diversity index, *s* = genus number, *ni* = number of individual genus of Collembola and *n* = total Collembola.

The criteria used was, H′ < 1.5: low diversity, 1.5 > H′ < 3.5: moderate diversity, H′ > 3.5: high diversity (Woiwod & Magurran 1990). The representation of individual in the taxa was evaluated using the Pielou Evenness Index (Pielou 1966) using the formula:


$$\text{E}={\text{H}}^{\prime }/\text{ln S}$$

where, E = evenness index, H′ = Shannon diversity index and S = number of genus.

All data were subjected to two-way analysis of variance (ANOVA) where experimental site or sampling time were used as repeated factors. The difference of treatment means was compared by least significant difference (LSD) at *P* = 0.05.

## RESULTS

### Experimental Locations Effect on Total Abundance and Diversity of Collembola

A total of 83,527 individual Collembola were captured and identified at the genus level, except for family of Tomoceridae, from four locations in West Java, as shown in Table 1. These Collembola belongs to 51 genera from 16 different families of 4 ordos. Most of them belongs to families of Sminthuridae (38.9%), Isotomidae (26.46%), Tomoceridae (18.04%) and Entomobrydae (10.01%), which accounted for 93.41%. As many as 17 out of 51 genera were common to all trial locations (*Acrocyrtus, Ascocyrtus, Entomobrya, Lepidocyrtus, Pseudosinella, Rambutsinella, Folsomia*, *Folsomina, Isotomiella, Isotomodes*, *Proisotoma, Subisotoma, Salina*, *Hypogastrura, Spaheridia, Pararrhopalites* and *Sphyrotheca*). As many as nine genera were found to be dominant, which account for 72.37% of all total individual Collembola captured, i.e., *Pararrhopalites* (Sminthuridae) (22%), *Sphyrotheca* (Sminthuridae) (16.9%), *Proisotoma* (Isotomidae) (13.87%), *Subisotoma* (Isotomidae) (5.91%), *Folsomia* (Isotomidae) (4.83%), *Acrocyrtus* (Entomobryidae) (3.07%), *Lepidocyrtus* (Entomobryidae) (2%), Xenylla (Hypogastruridae) (1.92%) and *Hypogastrura* (Hypogastruridae) (1.85%). Further analysis on these dominant genera showed that the abundance of *Proisotoma* was not different significantly in all experiment sites (see Table 2). All selected dominant Collembola, except *Proisotoma, Lepidocyrtus, Xenylla and Hypogastrura* were significantly more abundant in Sukamandi compared to those of the other experiment sites. Whereas all selected dominant Collembola except *Subisotoma* and *Xenylla* were present relatively in less number in Kuningan compared to those of the other locations.

Experiment locations significantly influenced the abundance, diversity index (H′), and evenness index (J) of Collembola (Table 2). The most abundant Collembola was observed in Sukamandi (in average of 3,662.44 ± 459.15 individual/plot), which was different significantly to those from Muara (2,572.33 ± 226.37 individual/plot), Banten (1,623.44 ± 311.53 individual/plot) and Kuningan (1,422.56 ± 66.32 individual/plot). Likewise, genus diversity in Kuningan and Muara with diversity indices of 1.58 and 1.51, were classified as moderate (Woiwod & Magurran 1990), higher than in Banten and Sukamandi with low diversity indices of 1.46 and 1.29, respectively. However, the composition of the Collembola community in Muara and Sukamandi were less even than in Kuningan and Banten, with Pielous evenness index of 0.55, 0.56, 0.66 and 0.68, respectively.

In Sukamandi, as many as 31 genera belong to 13 different families of 4 order were identified (Table 1). Three dominant genera were *Pararrhopalites* (Sminthuridae), *Sphyrotheca* (Sminthuridae) and *Folsomia* (Isotomidae) comprised of 25.24%, 20.4% and 11.71% of all identified Collembola, respectively. The numbers of other Collembola genera were found to be very few. Likewise, in Muara, *Sphyrotheca* (Sminthuridae), *Pararrhopalites* (Sminthuridae) and *Proisotoma* (Isotomidae) were dominant comprising of 25.27%, 20.2% and 12.6% of all captured Collembola, respectively. The Collembola population in Banten were dominated by *Proisotoma* (Isotomidae) (34.06%), *Pararrhopalites* (Sminthuridae) (16.08%), and *Lepidocyrtus* (Entomobryidae) (10.1%). While in Kuningan the population were dominated by *Pararrhopalites* (Sminthuridae) (23.66%), *Proisotoma* (Isotomidae) (16.45%), and *Subisotoma* (Isotomidae) (13.81%).

### Total Abundance and Diversity of Collembola During Rice Growth Stages

The diversity and abundance of Collembola were fluctuated during rice growths in all locations (Table 3). In Sukamandi and Muara, Collembola were significantly abundance at seedling stage, then decreased significantly at vegetative stage and increase in abundance to harvest. Whereas in Banten and Kuningan, Collembola were found less abundance at seedling stage and increased during generative stage and decreased in harvest time. In average from all experiment locations, however, the highest number of Collembola (3,331.44 ± 394.19) were trapped at seedling stage, while the lowest (1,027.11 ± 195.53) were collected at vegetative stage.

In general, at seedling stage, the Collembola communities were highly dominated by *Pararrhopalites*, *Sphyrotheca*, and *Folsomia* which accounted for 78.23% of total Collembola observed. All selected Collembola, except *Proisotoma*, decreased significantly in the vegetative stage. *Proisotoma* and *Pararrhopalites* dominated Collembola community at generative stage. Whereas at harvest time, almost all except *Sphyrotheca*, *Folsomia* and *Lepidocyrtus* were found in high number. *Proisotoma* was consistently found in high number at all stages of rice growth except at seedling stage. In contrast, *Pararrhopalites* was found to be highly abundant at seedling stage compared to those of other rice growth stages.

The Shannon’s diversity and evenness Pielous index, however, increased significantly from seedling to harvest stages. The lowest diversity and Pielous evenness index were observed at seedling stage (Table 3). The presence of certain genera (*Pararrhopalites* and *Sphyrotheca)* in high abundance (68%) has resulted in low diversity and evenness index. The abundance of both genera in the seedling stage seemed to be dependent on their microecosystem, which were was very wet, and also because they were still at their early emergent instar stages. Whereas the highest diversity and evenness were observed at harvest time, indicating that all genera were present in relatively more similar numbers.

### Effects of *Bt* and non-*Bt* Rice on Total Abundance and Diversity of Collembola

The abundances of Collembola were found to be similar between *Bt* and its wild type (non-*Bt*) rice cv Rojolele plots in all experiment sites (Table 4) and observation times (Table 5). The highest number of total Collembola individual was obtained in plot I (3,002.75 individual), followed by plot H (2,971.25 individual) which were planted with control rice cv IR42 and untransformed Rojolele without the application of pesticide, respectively. However, the abundance of Collembola both in *Bt* and its wild type cv Rojolele were not different statistically, indicating that this rice event had no detrimental effect to Collembola community. The application of pesticide Chlorantraniliprole (plot G), based on this experiment, did not significantly affect the abundance and the diversity index of Collembola (Table 4), this indicated that in this experiment, application of pesticide did not directly impact the Collembola communities.

The diversity of Collembola in the *Bt* and non-*Bt* plots in all experiment locations were also not statistically different (Table 4). The diversity of Collembola was, however, statistically different between the *Bt* and non-*Bt* plots at different growth stages (Table 5). The diversity of Collembola was found higher in all *Bt* plot except in plot D (H′ = 1.32) compared to non-*Bt* Rojolele (plot G) with H′ index of 1.33. Whereas the evenness index was significantly different among plots in each experiment sites (Table 4) indicating the presence of dominant Collembola, but not at different growth stages (Table 5).

Further analysis on selected dominant Collembola, including *Pararrhopalites*, *Sphyrotheca*, *Proisotoma*, *Folsomia*, *Hypogastrura*, *Lepidocyrtus*, *Subisotoma*, *Acrocyrtus* and *Xenylla* showed that their abundances in *Bt* and non-*Bt* plots were similar in all experiment sites (Table 6). Their abundances at different growth stages were also not statistically different (Table 7). These data indicated that *Bt* rice events as well as the non-*Bt* rice cv Rojolele caused no harmful effects on Collembola community.

## DISCUSSIONS

This is the first report on the assessment of the *Bt* rice impacts on the soil Collembola community during rice growth in the irrigated paddy field in Indonesia. From these confined field trials, we observed high abundance and diversity of Collembola community during rice growth. A total number of 83.527 individual Collembola from 4 ordos, 16 families and 51 genera were captured from the four confined field trials.

From this study, we found a high diversity of family and genus of Collembola in paddy fields planted with transgenic rice. Compared with previous study (Bai *et al*. 2010), which found three species of Collembola from three different families in rice fields in China, the diversity of Collembola in the rice field planted with *Bt* rice in West Java, Indonesia was more diverse. We observed the abundance of 4 out of 16 families identified, namely Sminthuridae, Isotomidae, Tomoceridae and Entomobrydae, which accounted for 93.41% of all Collembola captured. In other words, the abundances of the other nine families, including Cyphoderidae, Oncopoduridae, Paronellidae, Neelidae, Hypogastruridae, Neanuridae, Onychiuridae, Arrophalitidae, Bourletiellidae, Dicyrtomidae, Katiannidae and Sminthurididae, were comparatively lower. Those prominent families seemed to be common to rice paddy fields in Indonesia as shown by previous observations in paddy fields in Sumatera and Java islands (Indriyati & Wibowo 2008; Widyastuti 2005).

Genus diversity was obviously higher than that observed in the rice field in China (Bai *et al*. 2010). As many as 51 genus were identified from all experimental locations. Some of them were found to be common to all experiment locations, however some were more abundance at certain experiment sites (Tables 1 and 2). These differences might be related to the geographical and environmental conditions.

Interestingly, the presence of these Collembola fluctuated during the rice growth. Sminthuridae was more abundance during the wet season, whereas Entomobryidae and Isotomidae were more abundance during the dry or fallow periodes, similar to what were observed previously (Widyastuti 2005). The diversity and abundance of Collembola were greatly dependant on the food availability (litter quantity and variability), predator existence and environment factors including air temperature, rainfall level, field irrigation, soil humidity, soil texture, pH, soil C and N content (Bai *et al*. 2010; Indriyati & Wibowo 2008; Suhardjono *et al*. 2012; Warino *et al*. 2017; Widrializa *et al*. 2015). Field processing system and planting management were also affect the abundance of Collembola (Indriyati & Wibowo 2008). In rice and other monoculture crop system, the population was dominated only by few families or genera, thus the domination indices were relatively high while the diversities were low to medium with H′ (Shannon) indeces ranged from 1.0–1.9 (Indriyati & Wibowo 2008; Oktavianti *et al*. 2017; Widrializa *et al*. 2015), different to those in conserved forest, where they are more diverse and abundant (Oktavianti *et al*. 2017).

Thus, it is not surprising that the abundance and diversity of Collembola differ greatly from one experimental site to another and from planting to harvesting time. Number of Collembola in Sukamandi was significantly higher than those of the other three experiment sites, and dominated significantly by *Pararrhopalites* spp (Sminthuridae), Tomoceridae, *Spyrotheca* spp (Sminthuridae), Folsomia (Isotomidae), Proisotoma (Isotomidae) and Subisotoma (Isotomidae). Therefore, the Pielous evenness and the Shannon’s diversity index were significantly lower than those of the other three sites due to the presence of dominance genera (Table 2). Furthermore, *Pararrhopalites* spp. (Sminthuridae), *Spyrotheca* spp. (Sminthuridae), and *Folsomia* (Isotomidae) were significantly more abundant at seedling stage than the other growth stages. Whereas *Pararrhopalites* spp. (Sminthuridae), Proisotoma (Isotomidae) *Lepidocyrtus* (Entomobryidae) and *Tomoceridae* were found in greater number significantly at generative stage. Based on individual total number, Sminthuridae were found as the most dominant family during the observations which represents 35.92% of total Collembola trapped. Similar results were also previously observed in paddy fields where Sminthuridae family was found to be dominant both in the field and in the bund suggesting that Sminthuridae may play an important role in rice growing phase (Indriyati & Wibowo 2008; Widyastuti 2005). However, we observed that Isotomidae was also found in high number (25.94% of total Collembola trapped). Variations in the presence of dominant species might be caused by the presence of predators or due to the life cycle of the species itself. We also observed that *Proisotoma* (Isotomidae), interestingly, present in similar number in all plots, experimental sites and observation times except at seedling stage.

We conclude that there were no significant differences between *Bt*-rice and non-*Bt* rice cv Rojolele cultivations on Collembola abundance and diversity indices observed in all growth stages and experiment locations (see Tables 4 and 5). The results was consistent with similar experiment reported previously by Bai *et al*. (2010) using transgenic *Bt* rice expressing Cry1Ab protein on rice field in China, and other *Bt* crops containing different *Bt* genes (Arias-martín *et al*. 2016; Bitzer *et al*. 2005).

## CONCLUSION

The diversity and abundance of Collembola fluctuated during rice growth and were significantly different among all experimental locations in West Java, Indonesia. However, the diversity and abundance of Collembola on *Bt* rice plots were similar to those of the non-*Bt* rice cv Rojolele plots during rice growth. Further analysis on selected dominant Collembola, including *Pararrhopalites*, *Sphyrotheca*, *Proisotoma*, *Folsomia*, *Hypogastrura*, *Lepidocyrtus*, *Subisotoma*, *Acrocyrtus* and *Xenylla* which accounted for 72.37% of all total Collembola captured, showed that their abundance in *Bt* and non-*Bt* plots were not statistically different during rice growth in all experimental locations. Thus, based on these data it can be concluded that all six transgenic *Bt* rice events homozygous for *cry*1B-*cry*1Aa fusion genes had no effects on the biodiversity and abundance of Collembola communities.

## Figures and Tables

**Table 1:** Composition of Collembola at four confined field trials in West Java during 2012–2013.

Genus	Sukamandi		Muara		Banten		Kuningan		Total Individual

	N	D	N	D	N	D	N	D	
**Ordo:Entomobryomorpha**									
**Family: Cyphoderidae**									
Cyphoderopsis			27	0.117	15	0.103	42	0.328	84
**Family: Entomobryidae**									
Acrocyrtus	1,163	3.528	582	2.514	712	4.873	111	0.867	2,568
Alloscopus			60	0.259					60
Ascocyrtus	58	0.176	241	1.041	72	0.493	685	5.350	1,056
Coecobrya			44	0.190					44
Entomobrya	29	0.088	2	0.009	157	1.075	33	0.258	221
Heteromurus			10	0.043	3	0.021			13
Homidia	13	0.039					2	0.016	15
Lepidocyrtoides			63	0.272			7	0.055	70
Lepidocyrtus	15	0.046	46	0.199	1,476	10.102	134	1.047	1,671
Lepidosira			4	0.017			255	1.992	259
Pseudosinella	35	0.106	376	1.624	13	0.089	94	0.734	518
Rambutsinella	505	1.532	193	0.834	382	2.614	35	0.273	1,115
Seira			21	0.091	122	0.835	600	4.686	743
Sinella					1	0.007	5	0.039	6
Willowsia					1	0.007	1	0.008	2
**Family: Isotomidae**									
Desoria			109	0.471	1	0.007			110
Folsomia	3,861	11.713	12	0.052	151	1.033	14	0.109	4,038
Folsomides	8	0.024	42	0.181			18	0.141	68
Folsomina	267	0.810	52	0.225	65	0.445	26	0.203	410
Isotomiella	155	0.470	2	0.009	2	0.014	68	0.531	227
Isotomodes	19	0.058	1	0.004	179	1.225	2	0.016	201
Micrisotoma	44	0.133	4	0.017					48
Proisotoma	1,590	4.824	2,916	12.596	4,977	34.063	2,106	16.449	11,589
Pseudisotoma	469	1.423							469
Subisotoma	1,677	5.088	1,299	5.611	196	1.341	1,768	13.809	4,940
**Family: Oncopoduridae**									
Harlomillsia	9	0.027					2	0.016	11
Family: Paronellidae									
Bromocanthus			1	0.004	8	0.055	5	0.039	14
Callyntrura	5	0.015			14	0.096	4	0.031	23
Dicranocentroides	1	0.003							1
Salina	4	0.012	27	0.117	104	0.712	94	0.734	229
**Family: Tomoceridae**									
Tomoceridae	7,537	22.866	3,902	16.855	1,550	10.608	1,919	14.989	14,908
Tomocerus	1	0.003	41	0.177			118	0.922	160
**Ordo: Neelipleona**									
**Family:Neelidae**									
Megalothorax					1	0.007			1
Neelus	17	0.052	3	0.013					20
**Ordo: Poduromorpha**									
**Family:Hypogastruridae**									
Acherontiella			25	0.108					25
Hypogastrura	56	0.170	1,409	6.086	17	0.116	60	0.469	1,542
Xenylla			1,017	4.393	3	0.021	586	4.577	1,606
**Family: Neanuridae**									
Blasconura			15	0.065	2	0.014			17
Deuterobella			1	0.004					1
Neanuridae					1	0.007	1	0.008	2
Oudemansia			1	0.004					1
**Family: Onychiuridae**									
Protaphorura			2	0.009					2
Thalasaphorura	1	0.003							1
**Ordo: Symphypleona**									
**Family: Arrhopalitidae**									
Arrhopalites	2	0.006	72	0.311					74
Collophora					927	6.345	383	2.991	1,310
**Family: Bourletiellidae**									
Bourletiella	3	0.009							3
**Family: Dicyrtomidae**									
Dicyrtomidae							6	0.047	6
**Family: Katiannidae**									
Sminthurinus	1	0.003							1
**Family: Sminthurididae**									
Spaheridia	373	1.132	4	0.017	111	0,760	44	0.344	532
**Family: Sminthuridae**									
Pararrhopalites	8,320	25.241	4,676	20.198	2,350	16.084	3,029	23.659	18,375
Sphyrotheca	6,724	20.399	5,849	25.265	998	6.830	546	4.265	14,117

Total abundance	**32,962**	100	**23,151**	100	**14,611**	100	**12,803**	100	**83,527**

Total Order	4		4		4		3		
Total Family	13		12		11		12		
Total Genus	31		38		31		34		

*Notes*: Ecological indices: N = number of individual genus, D = dominance.

**Table 2:** Diversity and abundance of Collembola at different experiment sites.

Ecological indices	Sukamandi	Muara	Banten	Kuningan	F_3.01_	P_0.05_
Abundance of Collembola	3662.44 ± 459.15^c^	2572.33 ± 226.37^b^	1623.44 ± 311.53^a^	1422.56 ± 66.32^a^	12.98	3.07E-05

Abundance of selected dominant Collembola:						

Pararrhopalites	924.44 ± 163.59^b^	518.56 ± 119.14^a^	261.11 ± 40.63^a^	336.56 ± 34.51^a^	6.69	0.002
Sphyrotheca	747.11 ± 277.09^b^	649.89 ± 228.63^b^	110.89 ± 12.38^a^	33.33 ± 7.16^a^	4.02	0.019
Proisotoma	176.67 ± 46.13	324 ± 40.09	553 ± 247.36	234 ± 66.96	ns	ns
Subisotoma	186.33 ± 47.85^b^	144.33 ± 43.16^b^	21.78 ± 11.02^a^	196.44 ± 37.40^b^	3.72	0.025
Folsomia	429 ± 179.13^b^	1.33 ± 0.55^a^	16.78 ± 12.90^a^	1.56 ± 1.14^a^	5.55	0.005
Acrocyrtus	129.22 ± 30.14^c^	64.67 ± 10.14^b^	79.11 ± 8.12^b^	12.33 ± 4.95^a^	9.19	0.0003
Lepidocyrtus	1.67 ± 1.67^a^	5.11 ± 4.86^a^	164 ± 17.27^b^	14.89 ± 7.44^a^	67.20	8.04E-12
Hypogastrura	6.22 ± 2.55^a^	156.56 ± 45.05^b^	1.89 ± 0.72^a^	6.67 ± 2.60^a^	11.39	7.73E-05
Xenylla	0^a^	113 ± 39.13^b^	0.33 ± 0.24^a^	65.11 ± 11.70^b^	8.06	0.0007

Diversity indices:						

H′	1.29 ± 0.06^a^	1.51 ± 0.07^b^	1.46 ± 0.07^ab^	1.58 ± 0.08^b^	3.59	0.028
J	0.56 ± 0.02^ab^	0.55 ± 0.03^a^	0.68 ± 0.02^b^	0.66 ± 0.02^b^	11.22	8.54E-05

*Notes:* Mean ± Standard Error followed by different superscript lowercase letters within a row indicate significantly different at *P* = 0.05. H′ = Shannon index, J = Pielous evenness index, ns = not-significant at *P* = 0.05.

**Table 3:** Diversity and abundance of Collembola at different observation time during rice growth.

Ecological indices	Seedling	Vegetative	Generative	Harvest	*F*_3.01_	*P*_0.05_
Abundance of Collembola	3,331.44 ± 394.19^c^	1,027.11 ± 195.53^a^	2,443.11 ± 198.81^b^	2,479.11 ± 240.41^b^	16.03	6.23E-06
- Sukamandi	1,338.56 ± 290.93^b^	405.67 ± 100.074^a^	924.56 ± 136.20^ab^	993.67 ± 254.08^ab^	3.56	0.029
- Muara	1,503.67 ± 210.96^c^	86.89 ± 16.42^a^	91.56 ± 17.12^a^	890.22 ± 77.92^b^	36.92	3.75E-09
- Banten	280.67 ± 42.39^a^	327.11 ± 186.57^a^	885.11 ± 148.19^b^	130.56 ± 14.49^a^	9.49	0.0003
- Kuningan	208.56 ± 35.34^a^	207.44 ± 69.87^a^	541.89 ± 37.76^b^	464.67 ± 45.41^b^	10.59	0.0001

Abundance of selected dominant Collembola:						

-Pararrhopalites	1,001.11 ± 121.68^b^	252.11 ± 95.19^a^	472.22 ± 55.94^a^	315.22 ± 40.10^a^	14.38	1.44E-05
- Sphyrotheca	1,264.56 ± 327.48^b^	55.67 ± 13.58^a^	165.56 ± 29.18^a^	55.44 ± 14.29^a^	13.19	2.73E-05
- Proisotoma	48.56 ± 18.71^a^	326 ± 161.48^b^	505.89 ± 112.07^b^	407.22 ± 47.28^b^	4.94	0.008
- Subisotoma	28.89 ± 10.11^a^	25.5 6 ± 7.81^a^	59.22 ± 17.22^a^	430.33 ± 46.39^b^	55.21	6.4E-11
- Folsomia	340.67 ± 159.89^b^	77.22 ± 25.98^a^	0 ± 0^a^	12.44 ± 6.49^a^	4.15	0.017
- Acrocyrtus	48.44 ± 10.61^a^	26.56 ± 6.6^a^	27.78 ± 4.80^a^	182.56 ± 36.07^b^	14.82	1.14E-05
- Lepidocyrtus	0.11 ± 0.11^a^	12.11 ± 3.69^ab^	128.78 ± 14.38^c^	29.78 ± 9.6^b^	49.38	2.03E-10
- Hypogastrura	7.11 ± 2.79^a^	0.56 ± 0.34^a^	1.78 ± 0.98^a^	161.89 ± 45.71^b^	12.05	5.21E-05
-Xenylla	6.67± 1.36^a^	6.56 ± 2.28^a^	47.33 ± 11.62^a^	117.89 ± 40.76^b^	6.57	0.002

Diversity indices:						

H′	1.11 ± 0.05^a^	1.41 ± 0.08^b^	1.60 ± 0.03^c^	1.72 ± 0.04^c^	34.22	7.84E-09
J	0.52 ± 0.02^a^	0.64 ± 0.03^bc^	0.60 ± 0.02^b^	0.69 ± 0.02^c^	9.69	0.0002

*Notes:* Mean ± Standard Error followed by different superscript lowercase letters within a row indicate significantly different at *P* < 0.05. H′ = Shannon index, J′ = Pielous evenness index.

**Table 4:** Effects of *Bt* and non-*Bt* rice on abundance and diversity indices of Collembola in different experiment locations.

Rice lines	Ecological indices						

	Collembola abundance					H′	J

	Sukamandi	Muara	Banten	Kuningan	Total		
*Bt* :							
A	951.5 ± 103.85	420.25 ± 233.9	515.5 ± 278.03	355.5 ± 95.78	2,242.75 ± 537.4	1.43 ± 0.20	0.64 ± 0.04^bc^
B	454.5 ± 222.62	577 ± 265.34	399.5 ± 204.046	412.25 ± 130	1,843.25 ± 161.9	1.44 ± 0.13	0.65 ± 0.03^c^
C	871.25 ± 386.48	671 ± 339.79	311 ± 137.67	315.75 ± 88.33	2,169.00 ± 553.3	1.51 ± 0.26	0.60 ± 0.04^abc^
D	1,198.5 ± 433.58	970.75 ± 574.88	195.5 ± 75.85	447.5 ± 147.17	2,812.25 ± 923.8	1.32 ± 0.16	0.52 ± 0.06^a^
E	521.25 ± 90.31	620.75 ± 302.63	236.5 ± 77.41	389.5 ± 151.6	1,768.00 ± 333.1	1.52 ± 0.06	0.63 ± 0.01^bc^
F	619.75 ± 252.48	447.5 ± 191.3	259 ± 106.34	316 ± 106	1,642.25 ± 320.5	1.64 ± 0.10	0.66 ± 0.04^c^
Non-*Bt*:							
G	1121.5 ± 480	573.25 ± 307.07	413.25 ± 203.56	322.25 ± 72.62	2,430.25 ± 716.0	1.33 ± 0.23	0.56 ± 0.07^ab^
H	1,517.75 ± 467.88	767.25 ± 488.22	352.5 ± 206.56	333.75 ± 118.56	2,971.25 ± 1,108	1.57 ± 0.08	0.66 ± 0.05^c^
I	984.5 ± 406.49	740 ± 509.41	970 ± 455.57	308.25 ± 98.38	3,002.75 ± 631	1.37 ± 0.14	0.61 ± 0.03^bc^
*F*_2.36_	ns	ns	ns	ns	ns	ns	2.37
P_0.05_	ns	ns	ns	ns	ns	ns	0.049

*Notes:* Mean ± Standard Error followed by different superscript lowercase letters within a column indicate significantly.

**Table 5:** Effects of *Bt* and non-*Bt* rice on abundance and diversity indices of Collembola at different growth stage.

Rice lines	Ecological indices						

	Collembola abundance					H′	J

	Seedling	Vegetative	Generative	Harvest	Total		
*Bt* :							
A	748.25 ± 244.98	321.75 ± 168.46	703.75 ± 274.74	469 ± 141.12	2242.75 ± 401.9	1.43 ± 0.12^ab^	0.64 ± 0.02
B	515 ± 192.39	143 ± 53.13	675.5 ± 236.38	509.75 ± 196.26	1843.25 ± 450.82	1.44 ± 0.16^ab^	0.65 ± 0.06
C	947.75 ± 428.1	301.75 ± 160.31	483 ± 136.76	436.5 ± 247.88	2169.00 ± 562.1	1.51 ± 0.16a^bc^	0.60 ± 0.04
D	1259.5 ± 682.79	308.5 ± 159.35	466.25 ± 150.99	778 ± 263.01	2812.25 ± 838.25	1.32 ± 0.22^a^	0.52 ± 0.06
E	582.25 ± 249.59	147.75 ± 51	436.75 ± 108.7	601.25 ± 189.38	1768.00 ± 418.95	1.52 ± 0.15^abc^	0.63 ± 0.05
F	384.75 ± 95.23	101.25 ± 17.54	526 ± 152.99	630.25 ± 251.25	1642.25 ± 458.88	1.64 ± 0.09^c^	0.66 ± 0.03

Non-*Bt*:							
G	1071.25 ± 544.29	329.25 ± 160.27	614.5 ± 215.73	415.25 ± 145.77	2430.25 ± 662.82	1.33 ± 0.16^a^	0.56 ± 0.05
H	1102.5 ± 516.86	107 ± 37.27	900.5 ± 375.7	861.25 ± 469.48	2971.25 ± 873.71	1.57 ± 0.10^bc^	0.66 ± 0.06
I	884.5 ± 478.64	550.75 ± 417.15	690.75 ± 366.55	876.75 ± 422.18	3002.75 ± 321.18	1.37 ± 0.12^a^	0.61 ± 0.03
*F*_2.36_	ns	ns	ns	ns	ns	2.63	ns
P_0.05_	ns	ns	ns	ns	ns	0.03	ns

*Notes:* A: Rjl 04 F2.2 2.4-25-22-12-3-22, B: RFZ 3.2.2-1-6-28-1-10, C: RFZ 3.3.2A-11-25-12-5-3, D: RFZ 4.2.2-1-27-13-6-7, E: RFZ 4.2.3-28-15-2-8-20, F: RFZ 4.2.4-21-8-16-7-8, G: Wild-type parental rice cv Rojolele with insecticide, H: Wild-type parental rice cv Rojolele without pesticide application, and I: IR 42. Mean ± Standard Error followed by different superscript lowercase letters within a column indicate significantly different at *P* = 0.05. H′ = Shannon index, J = Pielous evenness index, ns = not-significant at *P* = 0.05.

**Table 6:** Effects of *Bt* and non-*Bt* rice on selected dominant Collembola in different experiment locations.

Rice lines	Collembola abundance (Mean ± Standard Error)								

	*Pararrhopalites*	*Sphyrotheca*	*Proisotoma*	*Folsomia*	*Hypogastrura*	*Lepidocyrtus*	*Subisotoma*	*Acrocyrtus*	*Xenylla*
*Bt* :									
A	527.75 ± 323.82	487.5 ± 244.89	314.75 ± 107.44	12.75 ± 11.43	8.75 ± 5.19	52 ± 33.83	114 ± 37.67	98.5 ± 42.86	94.5 ± 59
B	394.75 ± 36.89	113 ± 33.37	185.75 ± 35.51	26.25 ± 22.26	10.5 ± 7.26	70.25 ± 51.40	166 ± 92.81	49 ± 14.01	83.5 ± 67.58
C	691.75 ± 320.91	418 ± 374.79	214.25 ± 63.90	17.75 ± 16.44	11 ± 7.8	34 ± 32.02	112.5 ± 80.92	46 ± 11.97	39.5 ± 25.35
D	467 ± 274.26	688 ± 541.29	372.5 ± 127.78	338 ± 334.34	43.5 ± 41.17	35.25 ± 34.59	118.25 ± 60.06	81.5 ± 35.80	92.25 ± 61.95
E	381.5 ± 83.78	200 ± 137.59	143.25 ± 63.76	99.75 ± 98.42	112.25 ± 102.62	25.25 ± 25.25	155 ± 83.45	55.25 ± 18.25	24.5 ± 15.58
F	467.75 ± 180.42	111 ± 40.88	211.5 ± 60.84	38.5 ± 30.96	53.75 ± 52.09	36 ± 35.01	168 ± 92.20	52.25 ± 19.65	15.25 ± 8.83
Non-*Bt* :									
G	558 ± 206.53	864.5 ± 591.94	293 ± 107.14	10 ± 9.67	38.25 ± 29.86	43 ± 31.13	90.5 ± 17.94	43.75 ± 20.54	19 ± 16.14
H	497.25 ± 155.34	320.25 ± 128.91	348.75 ± 104.84	340.75 ± 337.09	77 ± 72.68	59 ± 52.76	196.5 ± 82.18	118 ± 62.70	19.75 ± 17.81
I	605.75 ± 209.19	265.5 ± 122.84	813.5 ± 554.93	125.75 ± 90.78	30.5 ± 28.18	63 ± 62.67	114.25 ± 52.81	97.75 ± 34.67	13.25 ± 9.78
*F*_2.36_	ns	ns	ns	ns	ns	ns	ns	ns	ns
*P* *_0.05_*	ns	ns	ns	ns	ns	ns	ns	ns	ns

*Notes:* A: Rjl 04 F2.2 2.4-25-22-12-3-22, B: RFZ 3.2.2-1-6-28-1-10, C: RFZ 3.3.2A-11-25-12-5-3, D: RFZ 4.2.2-1-27-13-6-7, E: RFZ 4.2.3-28-15-2-8-20, F: RFZ 4.2.4-21-8-16-7-8, G: Wild-type parental rice cv Rojolele with insecticide, H = Wild-type parental rice cv Rojolele without pesticide application, and I = IR 42. ns = not-significant at *P* = 0.05

**Table 7:** Effects of *Bt* and non-*Bt* rice on selected dominant Collembola at different growth stage.

Rice lines	Colembola abundance (Mean ± Standard Error)								

	*Pararrhopalites*	*Sphyrotheca*	*Proisotoma*	*Folsomia*	*Hypogastrura*	*Lepidocyrtus*	Subisotoma	Acrocyrtus	Xenylla
*Bt* :									
A	527.75 ± 129.33	487.5 ± 358.30	314.75 ± 134.34	12.75 ± 10.60	8.75 ± 7.44	52 ±33.69	114±42.79	98.5±65.68	94.5 ± 56.19
B	394.75 ± 187.17	113 ± 59.26	185.75 ± 76.62	26.25 ± 24.61	10.5 ± 8.91	70.25 ±43.11	166±111.13	49±23.43	83.5 ± 72.35
C	691.75 ± 302.26	418 ± 276.96	214.25 ± 101.03	17.75 ± 17.09	11 ± 8.04	34 ± 19.77	112.5 ± 86.53	46 ± 18.01	39.5 ± 23.30
D	467 ± 200.37	688 ± 607.81	372.5 ± 163.51	338 ± 244.25	43.5 ± 42.18	35.25 ± 26.28	118.25 ± 99.12	81.5 ± 65.58	92.25 ± 66.13
E	381.5 ± 158.61	200 ± 155.16	143.25 ± 83.71	99.75 ± 60.22	112.25 ± 108.26	25.25 ± 14.53	155 ± 104.89	55.25 ± 22.77	24.5 ± 11.20
F	467.75 ±157.16	111 ± 31.10	211.5 ± 40.62	38.5 ± 31.75	53.75 ± 52.43	36 ± 30.38	168 ± 163.01	52.25 ± 23.67	15.25 ± 6.09

Non-*Bt* :									
G	558 ± 118.24	864.5 ± 742.31	293 ± 118.39	10 ± 6.49	38.25 ± 29.93	43 ± 19.90	90.5 ± 55.06	43.75 ± 7.98	19 ± 8.53
H	497.25 ± 239.59	320.25 ± 208.91	348.75 ± 177.56	340.75 ± 303.92	77 ± 74.68	59 ± 43.76	196.5 ± 135.63	118 ± 68.13	19.75 ± 12.52
I	605.75 ± 317.18	265.5 ± 232.18	813.5 ± 335.38	125.75 ± 94.26	30.5 ± 26.35	63 ± 38.46	114.25 ± 96.36	97.75 ± 64.22	13.25 ± 6.7
*F*_2.36_	ns	ns	ns	ns	ns	ns	ns	ns	ns
P_0.05_	ns	ns	ns	ns	ns	ns	ns	ns	ns

*Notes:* A: Rjl 04 F2.2 2.4-25-22-12-3-22, B: RFZ 3.2.2-1-6-28-1-10, C: RFZ 3.3.2A-11-25-12-5-3, D: RFZ 4.2.2-1-27-13-6-7, E: RFZ 4.2.3-28-15-2-8-20, F: RFZ 4.2.4-21-8-16-7-8, G: Wild-type parental rice cv Rojolele with insecticide, H = Wild-type parental rice cv Rojolele without pesticide application, and I = IR 42. ns = not-significant at *P* = 0.05.

## References

[b1-tlsr-33-3-85] Arias-martín M, García M, José M, Ortego F, Castañera P, Farinós GP (2016). Agriculture, ecosystems and environment effects of three-year cultivation of Cry1Ab-expressing Bt maize on soil microarthropod communities. Agriculture, Ecosystems and Environment.

[b2-tlsr-33-3-85] Bai YY, Yan RH, Ye GY, Huang FN, Cheng JA (2010). Effects of transgenic rice expressing *Bacillus thuringiensis* Cry1Ab protein on ground-dwelling Collembolan community in postharvest seasons. Environmental Entomology.

[b3-tlsr-33-3-85] Bitzer RJ, Rice ME, Pilcher CD, Pilcher CL, Lam WKF (2005). Biodiversity and community structure of epedaphic and euedaphic springtails (Collembola) in transgenic rootworm Bt corn. Environmental Entomology.

[b4-tlsr-33-3-85] Bravo A (1997). Phylogenetic relationships of *Bacillus thuringiensis* δ-endotoxin family proteins and their functional domains. Journal of Bacteriology.

[b5-tlsr-33-3-85] Breitler JC, Vassal JM, Del Mar Catala M, Meynard D, Marfà V, Melé E, Royer M, Murillo I, San Segundo B, Guiderdoni E, Messeguer J (2004). Bt rice harbouring *cry* genes controlled by a constitutive or wound-inducible promoter: Protection and transgene expression under Mediterranean field conditions. Plant Biotechnology Journal.

[b6-tlsr-33-3-85] Chen Y, Tian JC, Shen ZC, Peng YF, Hu C, Guo YY, Ye GY (2010). Transgenic rice plants expressing a fused protein of Cry1Ab/Vip3H has resistance to rice stem borers under laboratory and field conditions. Journal of Economic Entomology.

[b7-tlsr-33-3-85] Estiati A, Herman M (2016). Biosafety regulation of genetically modified products in Indonesia. Analisis Kebijakan Pertanian.

[b8-tlsr-33-3-85] Filser J (2002). Role of Collembola in C and N cycling.

[b9-tlsr-33-3-85] Ho NH, Baisakh N, Oliva N, Datta K, Frutos R, Datta SK (2006). Translational fusion hybrid Bt genes confer resistance against yellow stem borer in transgenic elite Vietnamese rice (*Oryza sativa* L.) cultivars. Crop Science.

[b10-tlsr-33-3-85] Indriyati, Wibowo L (2008). Keragaman dan kemelimpahan Collembola serta Arthropoda tanah di lahan sawah organik dan konvensional pada masa bera. Jurnal Hama Dan Penyakit Tumbuhan Tropika.

[b11-tlsr-33-3-85] International Service for the Acquisition of Agri-biotech Applications (ISAAA) (2018). Global status of commercialized biotech/GM crops in 2018. ISAAA Brief No 54.

[b12-tlsr-33-3-85] Kumar S (2014). Biosafety issues of genetically modified organisms. Biosafety.

[b13-tlsr-33-3-85] Lavelle P, Spain AV (2001). Soil ecology.

[b14-tlsr-33-3-85] Lee Q, Widden P (1996). Folsomia candida, a “fungivorous” collembolan, feeds preferentially on nematodes rather than soil fungi. Soil Biology and Biochemistry.

[b15-tlsr-33-3-85] Liu Q, Hallerman E, Peng Y, Li Y (2016). Development of Bt rice and Bt maize in China and their efficacy in target pest control. International Journal of Molecular Sciences.

[b16-tlsr-33-3-85] Ludwig JA, Reynolds JF (1988). Statistical ecology: A primer on methods and computing (2).

[b17-tlsr-33-3-85] Makkar GS, Bentur JS, Arora R, Sandhu S (2017). Breeding for stem borer and gall midge resistance in rice. Breeding insect resistant crops for sustainable agriculture.

[b18-tlsr-33-3-85] Mendelsohn M, Kough J, Vaituzis Z, Matthews K (2003). Are Bt crops safe?. Nature Biotechnology.

[b19-tlsr-33-3-85] Nugroho S, Sari DI, Zahra F, Rachmawati S, Maulana BS, Estiati A (2021). Resistant performance of T10 Rojolele transgenic rice events harboring *cry1B*::*cry1Aa* fusion genes against the rice yellow stem borer *Scirpophaga incertulas* Wlk. IOP Conference Series: Earth and Environmental Science.

[b20-tlsr-33-3-85] Nugroho S, Estiati A, Slamet-Loedin IH, Gujar GT, Trisyono YA, Chen M (2021). Development of yellow stem borer resistant rice varieties in Indonesia. Genetically modified crops in asia pacific.

[b21-tlsr-33-3-85] Oktavianti R, Nurdin J, Herwina H (2017). Komunitas Collembola pada hutan konservasi dan perkebunan sawit di kawasan PT. Tidar Kerinci Agung (TKA), Sumatera Barat. Jurnal Biologi Universitas Andalas.

[b22-tlsr-33-3-85] Pielou EC (1966). The measurement of diversity in different types of biological collections. Journal of Theoretical Biology.

[b23-tlsr-33-3-85] Prakash D, Verma S, Bhatia R, Tiwary BN (2011). Risks and precautions of genetically modified organisms. International Scholarly Research Notices (ISRN) Ecology.

[b24-tlsr-33-3-85] Rahmawati S, Slamet-Loedin IH (2006). Introduction of cryIB-cryIAa hybrid gene into rice (*Oryza sativa*) genom cv. Rojolele using agrobacterium-mediated transformation. Hayati Journal of Biosciences.

[b25-tlsr-33-3-85] Settle WH, Whitten MJ (2000). The rule of small scale farmers in stengthening linkages between biodiversity and sustainable agriculture.

[b26-tlsr-33-3-85] Suhardjono YR, Deharveng L, Bedos A (2012). Biologi, ekologi, klasifikasi Collembola (ekor pegas).

[b27-tlsr-33-3-85] Usyati N, Buchori D, Manuwoto S (2009). Effectiveness of transgenic rice to the rice yellow stemborer *Scirpophaga incertulas* (Walker) (Lepidoptera: Crambidae). Jurnal Entomologi Indonesia.

[b28-tlsr-33-3-85] Visser S, Fitter AH, Atkinson D (1985). Role of the soil invertebrates in determining the composition of soil microbial communities. Biological interaction in soil.

[b29-tlsr-33-3-85] Warino J, Widyastuti R, Rahayuningsih Suhardjono Y, Nugroho B (2017). Keanekaragaman dan kelimpahan Collembola pada perkebunan kelapa sawit di Kecamatan Bajubang, Jambi. Jurnal Entomology Indonesia.

[b30-tlsr-33-3-85] Widrializa, Widyastuti R, Santosa DA, Djajakirana G (2015). The diversity and abundance of springtail (Collembola) on forests and smallholder in Jambi. Journal of Tropical Soils.

[b31-tlsr-33-3-85] Widyastuti R (2005). Population dynamics of microarthropods (Oribatida and Collembola) in rainfed paddy field ecosystem in Pati, Central Java. Jurnal Tanah dan Lingkungan.

[b32-tlsr-33-3-85] Woiwod IP, Magurran AE (1990). Ecological diversity and its measurement. Biometrics.

[b33-tlsr-33-3-85] Yaqoob A, Shahid AA, Samiullah TR, Rao AQ, Khan MAU, Tahir S, Mirza SA, Husnain T (2016). Risk assessment of Bt crops on the non-target plant-associated insects and soil organisms. Journal of the Science of Food and Agriculture.

